# Single parent status and children’s objectively measured level of physical activity

**DOI:** 10.1186/s40798-015-0020-1

**Published:** 2015-06-02

**Authors:** John Singhammer, Mathias Ried-Larsen, Niels Christian Møller, Peter Lund-Kristensen, Karsten Froberg, Lars Bo Andersen

**Affiliations:** 1 Department of Physiotherapy, VIA University College, Hedeager 2, DK8200 Aarhus, Denmark; 2 Institute of Sports Science and Clinical Biomechanics, Centre of Research in Childhood Health, University of Southern Denmark, Odense, Denmark

**Keywords:** Physical activity, Children, Adolescents, Family status, Meta-analysis

## Abstract

**Background:**

Single-parent family status has been investigated as a possible psychosocial determinant of children’s level of physical activity (PA)—although with mixed and inconclusive results. Prevailing evidence of the importance of two-parent family status as a resource for children’s PA is based on a mix of subjective and objective measurements of PA.

Objectives: To investigate if the level of PA among children living with a single parent was lower compared to children living with two parents by means of a meta-analysis of published and unpublished studies. We restricted our analysis to studies with objective measurements of PA.

**Methods:**

Data sources: The databases, Social Science Citation Index, PsycINFO, PubMed, and EBSCO were searched (1987–2013).

Study eligibility criteria: Observational studies comparing objectively measured PA between single-parent children and children from two-parent families.

Study appraisal and synthesis methods: We used guidelines from the *Cochrane Handbook of Systematic Reviews of Interventions* and a modified version of the Newcastle–Ottawa Scale overall to assess the quality of the included studies. We refrained from calculation of summary scores.

**Results:**

Twelve studies met the following inclusion criteria of which six were unpublished: (a) child age (6–18 years) and (b) objectively measured level of PA. Meta-analysis revealed pooled estimates of −0.01 for boys (95 % CI −0.04–0.03, *p* = 0.77, *I*^2^ = 6.5 %, *p* = 0.38) and 0.01 for girls (95 % CI −0.03–0.04, *p* = 0.62, *I*^2^ = 21.0 %, *p* = 0.24), respectively. Estimates show no differences in objectively measured physical activity between children living in single-parent families compared to children living with two parents. Analyses investigating seven potential moderators did not yield any statistical significant effect size estimates. No evidence of heterogeneity between studies was observed.

Limitations: Retrieved articles were assessed by several of the authors. Blinding of the authors was not feasible, as most of the authors have been involved in the studies.

**Conclusions:**

No evidence was found suggesting that children of single-parent families are in special need of extraordinary measures to facilitate their level of PA.

**Electronic supplementary material:**

The online version of this article (doi:10.1186/s40798-015-0020-1) contains supplementary material, which is available to authorized users.

## Key points

- Single-parent family status has been investigated as a possible determinant of children’s physical activity. The available studies that have used accelerometer-based measurements of physical activity are diverse and generally of poor quality.
- Pooled estimates show no differences in objectively measured physical activity between children living in single-parent families compared to children living with two parents.
- There is no evidence to conclude that children of single-parent families are in special need of extraordinary measures to facilitate their level of physical activity.

## Background

Children’s level of physical activity (PA) declines from adolescence [1] and remains low throughout adulthood for many individuals [2], thereby increasing the risk for later cardio-metabolic diseases (CMD) [3]. It has been suggested that children with single-parent family status (SPFS) are less physically active compared to children from dual-parent households [4, 5]. Studies investigating the association between family status and children’s PA are based on the hypothesis that children living with a single parent have less access to the beneficial entities of parental influence (e.g., role modeling and support) compared to children living in a two-parent home [4, 6–8]. However, past reviews have reported mixed findings, with some scholars reporting no evidence of an association between SPFS and children’s PA [9] and others reporting inconclusive findings [6]. With one out of every fourth child living with one parent in Europe and USA (24 and 25 %, respectively) [10, 11], SPFS may be an important factor that contributes to increased CMD risks through a low level of PA. An understanding of the social and contextual factors that influence PA is an important prerequisite for the design and implementation of PA-promoting interventions, and the association between SPFS and children’s PA has been of interest to scholars for decades [9, 6, 12, 13].

However, the methods used to evaluate the evidence of an association between SPFS and children’s level of PA may be flawed. For example, the vote-counting method used by Van der Horst et al. [13] and Sallis et al. [9] is based on the evaluation of (1) the number of studies included in their reviews, (2) the presence of a statistically significant association, and (3) consistency of the direction of the association in some, but not all of the included studies. One caveat of this approach is the lack of a quantification of the magnitude of evidence in terms of an overall effect size, which also takes the sample size of the included studies into account. As information of an overall effect size and the relative weight of each study included in the review (e.g., in terms of sample size) is not included in the study by Van der Horst et al. [13], the premise behind the reported conclusion is not transparent. More seriously, conclusions are based on a mixture of studies that measure children’s PA either objectively (e.g., by accelerometers) or subjectively by self-report. It has been argued that self-reported PA is vulnerable to culturally different interpretations, and hence, that the validity and reliability of objective measures of PA is superior to subjective measures [14]. Thus, comparison across studies that measures PA differently increases the possibility for misclassification of the outcome, which in turn may bias any estimate of an overall association between SPFS and children’s PA. Furthermore, conclusions on the SPFS-PA association do not include unpublished studies, which may otherwise change the bulk of available knowledge that is used to reach conclusions. The present study addresses these caveats by restricting the assessment of the SPFS-PA to studies that measure children’s PA objectively. Our overall objective was to investigate if the level of PA among children living with a single parent was lower compared to children living with two parents by means of a meta-analysis of results from published studies and from six studies that have not been published before. In line with the overall majority of studies investigating the association between SPFS and children’s PA, we report gender-separated results.

## Methods

We included cross-sectional studies and prospective observational studies with information on the level of PA for children, separately, by single- versus two-parent families. Inclusion criteria were studies comparing objectively measured PA between single-parent children and children from two-parent families. We explicitly choose studies that measured PA with accelerometers. Exclusion criteria were as follows: lacking information on parental status, insufficient information to calculate an effect size, self-reported PA estimates or cardio-respiratory fitness as the outcome measure (e.g., VO_2max_), and sedentary behavior or inactivity as the outcome measure. A protocol of this review was not registered prior to commencement.

The literature search was performed from March to November 2013. The literature search was limited to 1987 as studies using accelerometers for obtaining measures of PA conducted before 1987 are rare [15]. Participants were restricted to children aged 6 to 18 years, stratified by family status (single- versus two-parent families). In studies with estimates separated by age groups within family status, estimates were calculated separately for each group and combined to a single estimate as the number of studies available permitting calculation of age-separate effect size estimates were too low.

### Outcome

Objectively measured levels of PA were compared between children of single- and those of two-parent families and were either reported in tabular form or in the text.

### Search Strategy

The following electronic databases were searched: Social Sciences Citation Index [16], PsycINFO, PsycArticles, Journals OVID Full Text [17], Medline [18], and EBSCO [19], including the databases SportDiscus, CINAHL, Academic Search Premier, and ERIC. The following search terms were used: Physical activity, Accelerometry, Parents OR Family, Relations, Marital OR Status, Children OR Adolescents. The search term “psychosocial” was used with Journals OVID Full Text. Search terms including Boolean phrases were entered both separately and in combination. All terms were treated as separate blocks and were finally entered simultaneously using the separator AND. Duplicates across databases were identified with the EndNote software (version X4.0.2). The reference lists of primary studies and reviews identified from the literature search were scanned for other relevant studies. Searches were limited to studies published in English, Danish, Swedish, or Norwegian. This process revealed 16 studies suitable for investigating the association between SPFS and PA. Fourteen authors of these studies were contacted by email for information on unpublished studies, details of sampling methodology, sample size and attrition, and for further information necessary to compute effect sizes.

### Unpublished Studies

Raw data from the European Youth Heart Study (EYHS) from Denmark (1997 and 2003), Estonia (EYHS 1997), Portugal (EYHS 1997), and Norway (EYHS 1997) were available to the authors of the present study and were included for calculation of an overall effect size. Methods for the EYHS studies are reported elsewhere [20, 21].

Unpublished data from the Copenhagen School Child Intervention Study (CoSCIS) [22,23] were also available and included in the present analysis. A description of the methods, including sample size and measures of PA, are reported elsewhere [23].

Information on children’s PA, parental status, and mothers’ educational level was used for children aged from 9 to 15 years between 1997 and 1999 from the EYHS (1997, Denmark, Estonia, Portugal, and Norway *n* = 4515) and for 9-year-old children included in the Danish arm of the EYHS study in 2003 (*n* = 419) (Table 1). From CoSCIS, an analysis was conducted on 419 children with information on their level of PA and SPFS. Study protocols for the EYHS and CoSCIS studies conformed to international guidelines on biomedical research and were approved by the ethical board of University of Southern Denmark. The Danish Data Inspectorate approved the studies. The studies are conducted in accordance with the World Medical Association Declaration of Helsinki. Written informed consent was obtained from the children’s parent or legal guardian.

**Table 1:** Characteristic of studies included in the meta-analysis

Studies	*n*	Response rate (%)	Sampling strategy	Age	Percentage of single-parent families (%)	Publication status
Bagley et al. [27]	1215	35	STRS	7 and 11	13	Published
Bradley et al. [28]	1364	28	I/N	9	I/N	Published
Hesketh et al. [5]	2458	40	SRS	6 and 11	16	Published
Sallis et al. [30]	200	66	STRS	6 to 18	I/N	Published
Sallis et al. [29]	732	53	RA	10	22	Published
Sallis et al. [9]	297	40	I/N	9	13	Published
CoSCIS, Eiberg Hansen et al. [22]	411	52	CS	9	18	Unpublished
Riddoch et al. [20], EYHS Denmark, first cohort	1019	75	CRS	12	20	Unpublished
McMinn et al. [26], EYHS Denmark, second cohort	419	65	CRS	9	22	Unpublished
EYHS Estonia	1122	76	CRS	13	24	Unpublished
Riddoch et al. [20], EYHS Norway	754	75	CRS	12	21	Unpublished
Riddoch et al. [20], EYHS Portugal	1168	73	CRS	13	20	Unpublished
Total (%)	11,159	53	–	–	19	–

*SRS* simple random sample, *STRS* stratified random sample, *RA* random allocation as part of an intervention, *CS* convenience sample, *CRS* cluster randomized sample, *I*/*N* insufficient or no information

### Data Extraction

Forty-eight retrieved articles were independently assessed by two authors (JS and MRL). Of these, 12 studies (of which six were unpublished) were selected for further assessment. Blinding of the authors was not feasible, as most of the authors have been involved in the unpublished studies. We extracted the following characteristics: methods (study design, sampling strategy, sample size, location, publication status), participants (recruitment, gender, age, proportion of SPFS), and outcome (accelerometer model used, number of days measured, cut-point used to define level of measurement, percentage respondents with valid measurements). Any discrepancies were resolved with mutual agreement among reviewers.

### Assessing Risk of Bias

Prior to assessment, consensus was reached among all authors about the criteria for assessing risk of bias. We used the guidelines from the *Cochrane Handbook of Systematic Reviews of Interventions* [24] and the Newcastle–Ottawa Scale [25], although some criteria had to be modified for the specific topic. We assessed risk of bias by the following criteria: Selection (potential selection bias due to non-response, ascertainment of exposure), Comparability (adjustment for potential confounding factors (e.g., parental age and parental socioeconomic status)), and Outcome (incomplete outcome data with less than 75 % of the study sample, information on number of days assessing participant’s PA with accelerometers, length of epoch specified by the authors, and the cut-points used to define moderate to vigorous physical activity (MVPA)). The first author (JS) assessed the full text of the included studies and discussed the assessment with the other authors. Disagreement was resolved by consensus. Quality assessment was completed before data extraction was started. Currently, no evidence-based algorithm exists to quantify and evaluate the quality of the studies, and we refrained from calculation of summary scores that are often used to weight the studies in a later meta-analysis [24]. Hence, the quality of the included studies rest on our subjective assessment, and this may introduce selection bias to the meta-analysis. Therefore, all studies were used for analysis, irrespective of the quality assessment.

### Analyses

We used meta-analysis to calculate pooled gender-separated random effect size from all 12 studies. Effect size estimates were calculated for studies with detailed information on measurement of SPFS [5, 8, 20, 26] and for studies with adjustment for potential confounders [27, 28, 5, 8, 29]. Effect sizes were calculated for studies that assessed participant’s PA for at least 4 days [27, 28, 5, 23, 26, 20, 8], studies that obtained estimates of PA based on a 1-min epoch length [27, 28, 5, 26, 20, 22], studies that defined MVPA as 2000 counts.min^−1^ and above [26, 20], studies with valid outcome data of 75 % or more of the study sample [27, 28, 5, 26, 29], and unpublished studies [23, 26, 20]. We present pooled estimates expressed as Fisher’s *z*, which represents the direction and magnitude of the relation between SPFS and PA level. A positive effect size reflects a higher level of PA among children from single-parent families, compared to children from two-parent families. Calculation of the study-specific effect sizes was based on Pearson’s correlation coefficients or derivative statistics and sample sizes in three studies [8, 30, 29], *p* values and sample sizes in two studies [28, 5], and mean differences, standard deviations, and sample sizes in seven studies [20, 23, 27, 26]. Heterogeneity across the studies was evaluated with Higgins’ *I*^2^ statistics at an alpha level of 0.05.

We investigated for potential influence of bias on the pooled estimate by aspects of the study quality criteria. More specifically, we explored if our pooled estimates were influenced by the exclusion of studies with insufficient information on measurement of SPFS, studies that failed to control for potential confounders, studies with accelerometer data limited to less than 4 days, studies that used an epoch length of more than 1 min, studies based on a range of less than 2000 counts.min^−1^, studies with less than 75 % valid outcome data, and published data. This procedure is similar to a sensitivity analysis.

The percentage of respondents from single-parent families was calculated. Coding and descriptive analysis were conducted using the statistical software Stata (version 12.1) [31]. Calculation of effect size for each individual study, pooled estimates, and estimates of heterogeneity across studies was carried out using the software Comprehensive Meta Analysis (version 2.2.064) [32].

### Ethics

The manuscript does not contain clinical studies or patient data.

## Results

**Fig. 1:**
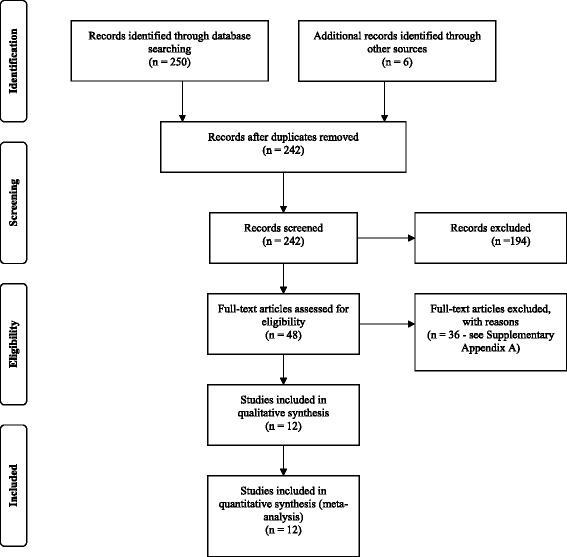
PRISMA flow diagram

The literature search revealed 250 published and six unpublished studies (Fig. 1). Of these, 48 studies were carefully inspected and references were obtained and assessed for inclusion. Twelve studies fulfilled the inclusion criteria of which six were unpublished (overall sample size = 11.159 children) (Table 1). Details of the 36 studies excluded and the reasons for exclusion are provided in Additional file 1. Of the 14 study authors contacted, two replied that they did not analyze their data separately by family status, as such measures were not obtained. One author no longer had access to the original data. The remaining authors did not respond.

Cross-sectional designs were used in all of the studies, except for the studies by Bradley et al. [28] and by Sallis et al. [29] who reported results from prospective cohort studies. The majority of the studies utilized some form of random allocation of study subjects. The CoSCIS study was based on a convenience sample [22]. Sallis et al. [30] did not report the sampling strategy, and information on the sampling strategy was insufficiently reported by Bradley et al. [28]. The age of the study participants ranged from 6 to 18 years. The percentage of children from single-parent families ranged from 13 to 24 %, and two studies did not report the percentage of children from single-parent families [8, 28]. Six studies were conducted in Europe [20, 22, 26], four in North America [30, 28, 8, 29], and two in Australia [27, 5]. The studies were conducted in community or school settings with primarily Caucasian samples. Measures of reliability were reported in five studies [30, 8, 28, 22, 29].

### Assessment of Risk of Bias

Overall, the quality of the studies differed considerably (Table 2). The response rate for the studies varied from 28 to 76 % (Table 1), and no study formally tested if respondents differed from non-respondents in terms of demographic factors. Seven studies provided information about how SPFS were measured [5, 8, 20] (Table 2). Estimates of PA, adjusted for potential confounding factors were provided in six studies [27, 28, 5, 30, 29, 8]. The number of days that accelerometer data were obtained varied considerably between the 12 included studies, but two studies used accelerometer data obtained for only 2 days [29, 30] (Table 3). Nine studies obtained estimates of PA based on a 1-min epoch [27, 28, 5, 26, 20, 22]. One study used counts.hour^−1^ to estimate PA [29], and two other studies by the same author did not report the epoch periods used to define PA [30, 8]. Definition of MVPA differed across studies. Four studies transformed accelerometer data to MET scores using the algorithm by Freedson et al. [33], and three studies defined MVPA as an activity occurring in the accelerometer range of 1017–3696 counts.min^−1^ [27, 28, 5]. Sallis et al. [30] used a PA level corresponding to six METS in one study and used the mean total counts as the outcome in two other studies [8, 29]. One study defined MVPA as an activity occurring of more than 1951 counts.min^−1^ [23] while the five studies from the EYHS defined MVPA as an activity occurring at 2000 or more counts.min^−1^ [26, 20] (Table 3). Valid accelerometer data was available for at least 75 % of the study sample in five studies [27, 28, 5, 29, 20].

**Table 2:** Assessment of study quality

Studies	Where results biased due to low response rate?	Was the exposure ascertained throughout?	Did authors adjust for confounding factors?	Was there valid outcome data for at least 75 % of the study sample?	Did authors report the number of days of PA assessment?	Did authors report the length of epoch?
Bagley et al. [27]	-	?	+	+	+	+
Bradley et al. [28]	-	?	+	+	+	+
Hesketh et al. [5]	-	+	+	+	+	+
Sallis et al. [30]	-	+	+	-	+	-
Sallis et al. [29]	-	-	+	+	+	+
Sallis et.al. [8]	?	?	+	-	+	-
Eiberg Hansen et al. [22] CoSCIS	-	?	-	-	+	+
Riddoch et al. [20], EYHS Denmark, first cohort	?	+	-	-	+	+
McMinn et al. [26], EYHS Denmark, second cohort	-	+	-	+	+	+
Riddoch et al. [20], EYHS Estonia	?	+	-	-	+	+
Riddoch et al. [20], EYHS Norway	?	+	-	-	+	+
Riddoch et al. [20], EYHS Portugal	?	+	-	-	+	+

“+” adequate information provided for assessment; “-” inadequate information provided for assessment; “?” no information provided

**Table 3:** Details of measurement of physical activity

Studies	Accelerometer model	Level of measurement used for calculation of effect size	Number of days (and hours) with PA assessment	Threshold, (counts.min^−1^ or counts.hour^−1^)		Cut-point used to define outcome	Percentage with valid measurements (%)
				Lower	Upper		
Bagley et.al. [27]	Manufacturing Technology Inc., Actigraph Model, AM7164-2.2C, USA	MVPA	8 days, day 2–6 included. At least four weekdays and one weekend day. At least 10 h per day	<10,000	>20,000,000	3–5.9 METS^a^ counts.min^−1^ (1017–3696)	97
Bradley et al. [28]	Computer Science and Applications (CSA), Inc., Shalimar, FL	MVPA	7 days, included for 4 days if non-zero counts from 5 a.m., and 60 min zero after 9 p.m., or 30 min zero after 10 p.m., or last zero count before 12 p.m.	I/N		3+ METS^a^ counts.min^−1^ (1017–3696)	75
Hesketh et al. [5]	Manufacturing Technology Inc. accelerometer, Model 7164	MVPA	6 days (24-h periods) included if complete data for at least 4 days	<10,000	>20,000,000	3+ METS^a^ counts.min^−1^ (1017–3696)	89
Sallis et.al. [30]	Computer Science and Applications (CSA), Inc., Shalimar, FL model 7164	VPA	7 days complete data included if no negative counts or “no long periods with zero counts”	I/N		6+ METS^a^	I/N
Sallis et al. [29]	Caltrac accelerometer (Hemokinetics, Inc., Madison, WI)	Mean total counts	From end of school day I to beginning of school day II, next morning	I/N		counts.hour^−1^	76
Sallis et.al. [8]^b^	Caltrac accelerometer (Hemokinetics, Inc., Madison, WI)	Mean total counts	From end of school day I to beginning of school day II, next morning or during a weekend (Friday to Monday morning)^c^	I/N		I/N	5
Eiberg Hansen et al. [22], CoSCIS	MTI Actigraph (Manufacturing Technology Inc., Fort Walton Beach, Florida, USA)	MVPA	4 days, including one weekend day, included if ≥600 min/day of activity for ≥3 days, and activity between 6 a.m. and 12 p.m. for at least 10 h.	<10,000	-	>1951 counts.min^−1^	69.7
Riddoch et al. [20], EYHS Denmark, first cohort	MTI Actigraph (Manufacturing Technology Inc., Fort Walton Beach, Florida, USA)	MVPA	5 days, including one weekend day, included if ≥600 min/day of activity for ≥3 days, and activity between 6 a.m. and 12 p.m. for at least 10 h.	<10,000	-	≥2000 counts.min^−1^	60
McMinn et al. [26], EYHS Denmark, second cohort	MTI Actigraph (Manufacturing Technology Inc., Fort Walton Beach, Florida, USA)	MVPA	5 days, including one weekend day, included if ≥600 min/day of activity for ≥3 days, and activity between 6 a.m. and 12 p.m. for at least 10 h.	<10,000	>20,000,000	≥2000 counts.min^−1^	85
Riddoch et al. [20], EYHS Estonia	MTI Actigraph (Manufacturing Technology Inc., Fort Walton Beach, Florida, USA)	MVPA	5 days, including one weekend day, included if ≥600 min/day of activity for ≥3 days, and activity between 6 a.m. and 12 p.m. for at least 10 h.	<10,000	-	≥2000 counts.min^−1^	52
Riddoch et al. [20], EYHS Portugal	MTI Actigraph (Manufacturing Technology Inc., Fort Walton Beach, Florida, USA)	MVPA	5 days, including one weekend day, included if ≥600 min/day of activity for ≥3 days, and activity between 6 a.m. and 12 p.m. for at least 10 h.	<10,000	-	≥2000 counts.min^−1^	44
Riddoch et al. [20], EYHS Norway	MTI Actigraph (Manufacturing Technology Inc., Fort Walton Beach, Florida, USA)	MVPA	5 days, including one weekend day, included if ≥600 min/day of activity for ≥3 days, and activity between 6 a.m. and 12 p.m. for at least 10 h.	<10,000	-	≥2000 counts.min^−1^	66

*MVPA* moderate to vigorous physical activity, *VPA* vigorous physical activity, *PA* physical activity, *I*/*N* insufficient or no information

^a^Calculated with algorithm from Freedson et al. [33]

^b^In the study by Sallis et al. [8], parents reported information on demographic, psychological, and biological circumstances. Data was collected by questionnaires that were distributed by 3648 students from seven schools. A total of 781 returned questionnaires were found complete and available for inclusion in further analysis (21 %). From the pool of 3648 children, 400 were selected at random and invited to wear an accelerometer to monitor the level of PA for 7 days. Accelerometer data for 265 children (66.3 %) were valid and included in further analysis. Of these, complete data from questionnaire and accelerometer was found among 200 (5 %) of the originally sampled 3648 children

^c^Activity during the school day was not measured

### Effect Sizes

Pooled estimates for all 12 studies were −0.01 for boys (95 % CI −0.04–0.03, *p* = 0.77, *I*^2^ = 6.5 %, *p* = 0.38) and 0.01 for girls (95 % CI −0.03–0.04, *p* = 0.62, *I*^2^ = 21.0 %, *p* = 0.24), respectively, where a positive effect size (Fisher’s *z*) favors single-parent status.

In an analysis of the seven studies with detailed information on measurement of SPFS, an effect size (Fisher’s *z*) of −0.02 was observed among boys (95 % CI −0.06–0.02, *p* = 0.35, *I*^2^ = 0 %, *p* = 0.55), and 0.00 among girls (95 % CI −0.05–0.06, *p* = 0.90, *I*^2^ = 45.8 %, *p* = 0.09), respectively.

Analysis of five studies that reported estimates adjusted for potential confounders yielded an effect size of −0.02 for boys (95 % CI −0.06–0.02, *p* = 0.34, *I*^2^ = 9 %, *p* = 0.41) and −0.01 for girls (95 % CI −0.05–0.03, *p* = 0.51, *I*^2^ = 0 %, *p* = 0.43), respectively.

Fisher’s *z* for 10 studies that obtained accelerometer data for 4 days or more was −0.01 for boys (95 % CI −0.05–0.02, *p* = 0.46, *I*^2^ = 0 %, *p* = 0.5) and 0.00 for girls (95 % CI −0.03–0.04, *p* = 0.85, *I*^2^ = 25.8 %, *p* = 0.90), respectively.

Effect size for nine studies that used a 1-min epoch length was −0.01 for boys (95 % CI −0.04–0.03, *p* = 0.66, *I*^2^ = 0 %, *p* = 0.5) and 0.01 for girls (95 % CI −0.02–0.05, *p* = 0.44, *I*^2^ = 0 %, *p* = 0.44), respectively.

Fisher’s *z* for five studies that defined MVPA as 2000 counts.min^−1^ and above was 0.01 for boys (95 % CI −0.05–0.07, *p* = 0.73, *I*^2^ = 0 %, *p* = 0.6) and 0.03 for girls (95 % CI −0.04–0.09, *p* = 0.47, *I*^2^ = 39.7 %, *p* = 0.16), respectively.

The analysis of five studies with valid outcome data for 75 % or more of the study sample revealed an effect size of −0.00 among boys (95 % CI −0.05–0.05, *p* = 0.89, *I*^2^ = 12.5 %, *p* = 0.33) and 0.00 among girls (95 % CI −0.03–0.05, *p* = 0.68, *I*^2^ = 0 %, *p* = 0.88), respectively.

Six studies were unpublished. Effect size was −0.00 for boys (95 % CI −0.05–0.05, *p* = 0.99, *I*^2^ = 0 %, *p* = 0.6) and 0.03 for girls (95 % CI −0.03–0.09, *p* = 0.32, *I*^2^ = 27.7 %, *p* = 0.23), respectively.

As the effect size was close to zero in all analyses, and since evidence of statistically significant variability in effect size across studies was lacking, no further attempt to investigate for bias was warranted.

## Discussion

The results of the present meta-analysis show no difference in the level of PA between children living in single-parent families and those living in two-parent families. Thus, the findings indicate that SPFS is not associated with children’s level of PA. The overall quality of the studies was less than optimal. Most of the published studies had a low response rate, and the majority of the studies were based on small samples. As the reported percentages of children exposed to SPFS were below what is officially reported for Europe [10] and USA [11], it may suggest that respondents from single-parent families tended to participate in the studies to a lower extent than what could be expected from random sampling variability. However, none of the included studies addressed this issue. Thus, the low percentage of children from single-parent families suggests that most of the studies may be influenced by selection bias.

With regard to the outcome (accelerometer-based assessment of PA) several potential sources of bias was observed. For example, monitoring for at least 4 days has been argued as necessary to obtain reliable measurements of PA among children and adolescents [34]. In the studies included in the present analysis, monitoring varied between 1 and 8 days and the daily average level of PA estimated in studies with less than 4 days of monitoring may not reflect the participant’s level of PA. Also, it has been argued that the use of a 1-min epoch length to determine the amount of time spent in different levels of PA intensity fails to capture the time children and adolescents spent in vigorous activity [35]. The majority of the included studies used a 1-min epoch length, and this may have caused underestimation of the time spent in MVPA among participants. Hence, the studies may have failed to capture differences in time spent in vigorous PA by children’s family status. Interestingly, the early study by Sallis et al. [30] was confined to vigorous PA and was the only study that showed a higher level of PA among single-parent children. Definition of MVPA differed between the studies, and this reflects the ongoing debate about the most appropriate cut-point for defining intensities of accelerometer-based assessments of PA [36]. However, the level of PA was equal across SPFS in all studies but one, and the discussion about an ideal cut-point may be less relevant for results of the present analysis. A minority of the studies had valid accelerometer data for at least 75 % of the study sample. The availability of valid accelerometer data from 5 % of the sample in the study by Sallis et al. [30] is especially notable. As in that study, poor compliance of the participants to wear the accelerometers, malfunction of the devices or loss of information because of study design, may be responsible for the low availability of data. Consequently, studies with a low proportion of valid outcome data may be prone to selection bias, and as information on the family status of the participants with missing accelerometer data is rarely provided, comparability of the study results may be hampered. Thus, our results should be treated with caution.

Our results are at odds with the results of previous reviews. For example, Sallis et al. [9] concluded that single-parent status was indeterminately related to children’s PA. In this respect, a clearer distinction between single-parent family structures (e.g., distinction between divorced and widowed) has been called for as failure to do so could lead to comparison across different family environments that may not be comparable [6]. Similarly, potential differences between children who experience a true SPFS and those who live in single-parent family but have dual custody/regular contact with a second parent may also influence the comparability of results. Our data did not permit us to explicitly address this issue. However, measurements of SPFS are almost always self-reported and encompass a range of family structures that are often reduced to broader categories (e.g., single/two parent) in the final analysis. The self-reported measures of family status include children with and without regular contact with a second parent. As the effect sizes between and within studies in the present meta-analysis are homogeneous, it is difficult to see how further refinement of the SPFS measurement could alter the results.

Detrimental consequences of SPFS include economic difficulties and deterioration of social networks [37]. Such consequences have been observed to exert a substantial influence on children’s cognitive and emotional development and well-being later in life, through various pathways [38]. Elder [38] proposed that the key to the successful development of children exposed to adverse social conditions in childhood was their parents’ ability to adequately face a difficult situation and act within the existing opportunities and constraints to counteract the child’s experience of a troubled time. Others have supported this notion [39]. Thus, it is possible that single parents may successfully allocate the resources necessary to avoid restricting opportunities for children’s PA and imply that the single parent try to compensate for the shortage of support by the absence of a second partner. Although these explanations are only suggestive, the findings of the present meta-analysis indicate at least that single-parent status does not inhibit children’s level of PA. However, the findings do not give any indication of possible differences in patterns or intensity of PA among children, separately by family status, although such differences have been reported [4, 30, 5]. Similarly, it is possible that participation in leisure activities and membership in sports clubs may be different for children of single-parent families to those from two-parent families. These associations have not been adequately addressed in the research literature, although some tentative results do exist [40, 7]. For example, Lindquist et al. [7] observed a higher level of participation in sports activities for SPFS children but a lower level of participating in PE classes in school. These findings may be somewhat contradictory but highlight the complexity of the composition of PA among children and underscore the need for developing measurement tools that enables a detailed monitoring of the total level of PA among children, regardless of their sociocultural background.

SPFS is assumed to be a proxy for several psychosocial factors important to children’s PA, with instrumental support as the most prominent [30, 9, 28, 27, 5]. It is also assumed that single-parent families differ from two-parent families in their lower level of role modeling abilities and financial capacity [41]. The results of the present analysis dismiss evidence of a differential level of PA among children from single-parent families, and the results may suggest that the assumed disadvantaged fostering milieu for PA in single-parent families is not warranted. A more subtle relation between SPFS, presence of siblings, and children’s PA was suggested by Bagley et al. [27], who reported a higher level of PA for boys with an older male sibling in single-parent families and for girls growing up with siblings regardless of gender. Similarly, Duncan et al. [42], suggested that the level of PA is interdependent among siblings but not among children and parents. Children of single parents may experience less parental support for engaging in PA or lack access to observational learning situations than children of two-parent families, but may compensate for the lack of parental involvement and stimuli and seek other sources of information and role models that assist in shaping their PA level. Siblings may be one such source of information, as well as role models.

### Strengths and Limitations

The inclusion of six unpublished studies strengthens the present meta-analysis by allowing a detailed inspection of variability across diverse study settings and geographical locations. Another strength was our decision to restrict the analysis to studies that used accelerometry to measure children’s PA permitted a detailed evaluation of the various decisions in managing accelerometer data on PA.

Some limitations should be mentioned. The search strategy used for the present study may have been too restrictive. Firstly, only English, Danish, Swedish, and Norwegian language studies were included. Secondly, efforts to locate unpublished literature were restricted to contacting authors of published studies to request information on family status and searching four databases, although several more may exist [43]. Thirdly, assessment of risk of bias in the included studies was not blinded. However, a recent meta-analysis suggested that the effectiveness of blinding authors in bias assessment is unclear and may be redundant [44]. Fourthly, the word “accelerometry” (ACELLEROM*) may have restricted the search to studies based on PA measured with accelerometric methods, although other methods exist (e.g., doubly labeled water [45], measure of heart rate, and indirect calorimetry). It has been suggested that measurement of PA using various electronic devises may not be comparable due to variability in obtaining accurate measures of energy expenditure—the basic component of PA [1]. If so, our restricted search may have revealed studies that are comparable, at least with respect to PA. Nevertheless, the search term “accelerometry” automatically includes related terms such as “pedometer”, at least in Medline [18]. A search replacing the word “accelerom”* with the MeSH term “actigraphy” revealed 15 studies, of which 10 were not previously identified but deemed irrelevant as they fell into our exclusion criteria. This suggests that the chosen search strategy was sufficient to capture relevant studies. Fifthly, we combined effect size estimates of age groups into a single estimate and this may have concealed variability in levels of PA for younger versus older children. However, we assessed the differences in PA among children aged 9 and 15 years old living in single- versus two-parent families using the data from the EYHS, and results were virtually identical to analysis with combined age groups (results not shown). Hence, our combination of estimates for age groups does not impede with the overall results. Finally, our results are obtained from studies conducted in developed countries. The countries represented may share common sociocultural characteristics that shape the SPFS–PA relationship, albeit this was not investigated. Restricting our analysis to studies that share similar sociocultural characteristics provides us with a better basis for comparing results. On the other hand, it may also restrict our ability to generalize the overall conclusions to developing countries. Hence, our results may only apply for children and families in developed countries.

## Conclusions

The present meta-analysis shows that single-parent status is not associated with children’s level of PA. However, the included studies are heavily biased. Hence, caution should be exercised in interpretation of the overall result. Nevertheless, no evidence exists suggesting that children of single-parent families are in special need of extraordinary measures to facilitate their level of PA.

## Additional file

Additional file 1:
**Studies excluded for meta-analysis of single-parent family status and children’s level of physical activity.**
