# Supplementary material for: Single parent status and children’s objectively measured level of physical activity
Source: Sports Med Open. 2015 Jun 2;1:10. doi: 10.1186/s40798-015-0020-1 (PMC4532699; doi:10.1186/s40798-015-0020-1)
Supplement: Additional file 1: — Studies excluded for meta-analysis of single-parent family status and children’s level of physical activity. [file 40798_2015_20_MOESM1_ESM.docx]

Additional file 1: Studies excluded for meta-analysis of single parent family status and children’s level of physical activity

| *No.* | *Author* | *Reason for exclusion* |
| --- | --- | --- |
| 1 | Hhd NT, (2012) [[1](#_ENREF_1)] | *No information on family status* |
| 2 | Sharifah WW, Nur HH, Ruzita AT, Roslee R, Reilly JJ. (2011) [[2](#_ENREF_2)] | *No information on family status* |
| 3 | Finni, T., et al., (2011) [[3](#_ENREF_3)] | *Protocol for intervention study* |
| 4 | Taylor, B.J., et al., (2011) [[4](#_ENREF_4)] | *Intervention on mothers of toddlers* |
| 5 | Salmon, J., et al., (2011) [[5](#_ENREF_5)] | *Protocol for intervention study* |
| 6 | Wilson, D.K., et al., (2011) [[6](#_ENREF_6)] | *No information on family status* |
| 7 | Olvera, N., et al., (2011) [[7](#_ENREF_7)] | *No information on family status* |
| 8 | Cleland, V., et al., (2011) [[8](#_ENREF_8)] | *No information on family status* |
| 9 | Nyberg, G., et al., (2011) [[9](#_ENREF_9)] | *Protocol for intervention study* |
| 10 | Dunton, G.F., et al., (2011) [[10](#_ENREF_10)] | *No information on family status* |
| 11 | Fuemmeler, B.F., C.B. Anderson, and L.C. Masse (2011) [[11](#_ENREF_11)] | *No information on family status* |
| 12 | Jago, R., et al., (2011) [[12](#_ENREF_12)] | *No information on family status* |
| 13 | Kitzman-Ulrich, H., et al., (2010) [[13](#_ENREF_13)] | *No information on family status* |
| 14 | Fisher, A., et al., (2011) [[14](#_ENREF_14)] | *No information on family status* |
| 15 | Mattocks, C., et al., (2008) [[15](#_ENREF_15)] | *No information on family status* |
| 16 | Carver, A., et al., (2010) [[16](#_ENREF_16)] | *No information on family status* |
| 17 | Veitch, J., J. Salmon, and K. Ball, (2010) [[17](#_ENREF_17)] | *No information on family status* |
| 18 | Davison, K.K. and R. Jago, (2009) [[18](#_ENREF_18)] | *No information on family status* |
| 19 | Magnusson, K.T., et al., (2008) [[19](#_ENREF_19)] | *No information on family status* |
| 20 | Salmon, J., et al., (2005) [[20](#_ENREF_20)] | *No information on family status* |
| 21 | Beunen G.P., et al., [[21](#_ENREF_21)] | *No information on family status* |
| 22 | Hume, C., J. Salmon, and K. Ball, (2005) [[22](#_ENREF_22)] | *Qualitative study* |
| 23 | Kalakanis, L.E., et al., (2001) [[23](#_ENREF_23)] | *No information on family status* |
| 24 | Trost, S.G., et al., (2001) [[24](#_ENREF_24)] | *No information on family status* |
| 25 | Sallis, J.F., T.L. McKenzie, and J.E. Alcaraz, (1993) [[25](#_ENREF_25)] | *No information on family status* |
| 26 | Freedson, P.S. and S. Evenson, (1991) [[26](#_ENREF_26)] | *No information on family status* |
| 27 | Moore, L.L., et al., (1991) [[27](#_ENREF_27)] | *No information on family status* |
| 28 | Bender, J.M., et al., (2005) [[28](#_ENREF_28)] | *No information on family status* |
| 29 | Martin-Matillas, M., et al., (2011) [[29](#_ENREF_29)] | *No information on family status* |
| 30 | Morgan, P.J., et al., (2008) [[30](#_ENREF_30)] | *No information on family status* |
| 31 | Beech, B. M., Kumanyika, S. K., Baranowski, T., Davis, M., Robinson, T. N., Sherwood, N. E., et al., (2004) [[31](#_ENREF_31)] | *No information reported separately for family status* |
| 32 | Gustafson, S. L., & Rhodes, R. E. (2006) [[32](#_ENREF_32)] | *Review article* |
| 33 | Sallis, J., Prochaska, J., & Taylor, W. (2000) [[33](#_ENREF_33)] | *Review article* |
| 34 | Ferreira, I., van der Horst, K., Wendel-Vos, W., Kremers, S., van Lenthe, F. J., & Brug, J. (2006) [[34](#_ENREF_34)] | *Review article* |
| 35 | Crawford, D., Cleland, V., Timperio, A., Salmon, J., Andrianopoulos, N., Roberts, R., et al. (2010) [[35](#_ENREF_35)] | *Similar data as Hesketh et al.,[*[*36*](#_ENREF_36)*]* |
| 36 | McMinn, A. M., Van Sluijs, E. M. F., Wedderkopp, N., Froberg, K., & Griffin, S. J. (2008) [[37](#_ENREF_37)] | *Data derived from EYHS [*[*38*](#_ENREF_38)*] but with insufficient information for calculation of an effect size* |

**References**

1. Hhd NT, K HT, Van der Ploeg HP, Hardy LL, Kelly PJ, Dibley MJ. Longitudinal Physical Activity Changes In Adolescents: Ho Chi Minh City Youth Cohort. Medicine and science in sports and exercise. 2012. doi:10.1249/MSS.0b013e31824e50dc.

2. Sharifah WW, Nur HH, Ruzita AT, Roslee R, Reilly JJ. The Malaysian Childhood Obesity Treatment Trial (MASCOT). Malays J Nutr. 2011;17(2):229-36.

3. Finni T, Saakslahti A, Laukkanen A, Pesola A, Sipila S. A family based tailored counselling to increase non-exercise physical activity in adults with a sedentary job and physical activity in their young children: design and methods of a year-long randomized controlled trial. Bmc Public Health. 2011;11:944. doi:10.1186/1471-2458-11-944.

4. Taylor BJ, Heath AL, Galland BC, Gray AR, Lawrence JA, Sayers RM et al. Prevention of Overweight in Infancy (POI.nz) study: a randomised controlled trial of sleep, food and activity interventions for preventing overweight from birth. Bmc Public Health. 2011;11:942. doi:10.1186/1471-2458-11-942.

5. Salmon J, Arundell L, Hume C, Brown H, Hesketh K, Dunstan DW et al. A cluster-randomized controlled trial to reduce sedentary behavior and promote physical activity and health of 8-9 year olds: the Transform-Us! study. Bmc Public Health. 2011;11:759. doi:10.1186/1471-2458-11-759.

6. Wilson DK, Lawman HG, Segal M, Chappell S. Neighborhood and Parental Supports for Physical Activity in Minority Adolescents. American Journal of Preventive Medicine. 2011;41(4):399-406. doi:10.1016/j.amepre.2011.06.037.

7. Olvera N, Smith DW, Lee C, Liu J, Lee J, Kim JH et al. Comparing high and low acculturated mothers and physical activity in Hispanic children. Journal of physical activity & health. 2011;8 Suppl 2:S206-13.

8. Cleland V, Timperio A, Salmon J, Hume C, Telford A, Crawford D. A longitudinal study of the family physical activity environment and physical activity among youth. Am J Health Promot. 2011;25(3):159-67. doi:10.4278/ajhp.090303-QUAN-93.

9. Nyberg G, Sundblom E, Norman Å, Elinder LS. A healthy school start-Parental support to promote healthy dietary habits and physical activity in children: Design and evaluation of a cluster-randomised intervention. Bmc Public Health. 2011;11(1):185-91. doi:10.1186/1471-2458-11-185.

10. Dunton GF, Liao Y, Intille S, Wolch J, Pentz MA. Physical and social contextual influences on children's leisure-time physical activity: an ecological momentary assessment study. Journal of physical activity & health. 2011;8 Suppl 1:S103-8.

11. Fuemmeler BF, Anderson CB, Masse LC. Parent-child relationship of directly measured physical activity. Int J Behav Nutr Phys Act. 2011;8:17. doi:10.1186/1479-5868-8-17.

12. Jago R, Davison KK, Brockman R, Page AS, Thompson JL, Fox KR. Parenting styles, parenting practices, and physical activity in 10- to 11-year olds. Preventive Medicine. 2011;52(1):44-7. doi:10.1016/j.ypmed.2010.11.001.

13. Kitzman-Ulrich H, Wilson DK, Van Horn ML, Lawman HG. Relationship of body mass index and psychosocial factors on physical activity in underserved adolescent boys and girls. Health Psychol. 2010;29(5):506-13. doi:10.1037/a0020853.

14. Fisher A, Saxton J, Hill C, Webber L, Purslow L, Wardle J. Psychosocial correlates of objectively measured physical activity in children. Cancer Research UK Health Behaviour Research Centre, Department of Epidemiology and Public Health, University College London, London, UK, (C) European Journal of Public Health 2011. Published by Oxford University Press. All rights reserved. 2011. <http://ovidsp.ovid.com/ovidweb.cgi?T=JS&PAGE=reference&D=ovftl&NEWS=N&AN=00060993-201103000-00006>. Accessed d0w, 9204966 21.

15. Mattocks C, Deere K, Leary S, Ness A, Tilling K, Blair SN et al. Early life determinants of physical activity in 11 to 12 year olds: cohort study. British journal of sports medicine. 2008;42(9):721-4.

16. Carver A, Timperio A, Hesketh K, Crawford D. Are children and adolescents less active if parents restrict their physical activity and active transport due to perceived risk? Social Science & Medicine. 2010;70(11):1799-805. doi:10.1016/j.socscimed.2010.02.010.

17. Veitch J, Salmon J, Ball K. Individual, social and physical environmental correlates of children's active free-play: a cross-sectional study. Int J Behav Nutr Phys Act. 2010;7:11. doi:10.1186/1479-5868-7-11.

18. Davison KK, Jago R. Change in Parent and Peer Support across Ages 9 to 15 yr and Adolescent Girls' Physical Activity. Medicine and science in sports and exercise. 2009;41(9):1816-25. doi:10.1249/MSS.0b013e3181a278e2.

19. Magnusson KT, Sveinsson T, Arngrimsson SA, Johannsson E. Predictors of fatness and physical fitness in nine-year-old Icelandic school children. Int J Pediatr Obes. 2008;3(4):217-25. doi:10.1080/17477160802169482.

20. Salmon J, Timperio A, Telford A, Carver A, Crawford D. Association of family environment with children's television viewing and with low level of physical activity. Obesity Research. 2005;13(11):1939-51. doi:10.1038/oby.2005.239.

21. Beunen GP, Lefevre J, Philippaerts RM, Delvaux K, Thomis M, Claessens AL et al. Adolescent correlates of adult physical activity: a 26-year follow-up. Medicine and science in sports and exercise. 2004;36(11):1930-6.

22. Hume C, Salmon J, Ball K. Children's perceptions of their home and neighborhood environments, and their association with objectively measured physical activity: a qualitative and quantitative study. Health Education Research. 2005;20(1):1-13.

23. Kalakanis LE, Goldfield GS, Paluch RA, Epstein LH. Parental activity as a determinant of activity level and patterns of activity in obese children. Res Q Exerc Sport. 2001;72(3):202-9.

24. Trost SG, Kerr LM, Ward DS, Pate RR. Physical activity and determinants of physical activity in obese and non-obese children. International Journal of Obesity & Related Metabolic Disorders. 2001;25(6):822.

25. Sallis JF, McKenzie TL, Alcaraz JE. Habitual physical activity and health-related physical fitness in fourth-grade children. Am J Dis Child. 1993;147(8):890-6.

26. Freedson PS, Evenson S. Familial aggregation in physical activity. / Le rassemblement familial dans l ' activite physique. Research Quarterly for Exercise & Sport. 1991;62(4):384-9.

27. Moore LL, Lombardi DA, White MJ, Campbell JL, Oliveria SA, Ellison RC. Influence of parents' physical activity levels on activity levels of young children. J Pediatr. 1991;118(2):215-9.

28. Bender JM, Brownson RC, Elliott MB, Haire-Joshu DL. Children's physical activity: using accelerometers to validate a parent proxy record. Medicine and science in sports and exercise. 2005;37(8):1409-13.

29. Martin-Matillas M, Ortega FB, Ruiz JR, Martinez-Gomez D, Marcos A, Moliner-Urdiales D et al. Adolescent's physical activity levels and relatives' physical activity engagement and encouragement: the HELENA study. Eur J Public Health. 2011;21(6):705-12. doi:10.1093/eurpub/ckq143.

30. Morgan PJ, Okely AD, Cliff DP, Jones RA, Baur LA. Correlates of objectively measured physical activity in obese children. Obesity (Silver spring). 2008;16(12):2634-41. doi:10.1038/oby.2008.463.

31. Beech BM, Kumanyika SK, Baranowski T, Davis M, Robinson TN, Sherwood NE et al. Parental cultural perspectives in relation to weight-related behaviors and concerns of African-American girls. Obesity Research. 2004;12 Suppl:7S-19S. doi:10.1038/oby.2004.264.

32. Gustafson SL, Rhodes RE. Parental correlates of physical activity in children and early adolescents. Sports Medicine. 2006;36(1):79-97.

33. Sallis J, Prochaska J, Taylor W. A review of correlates of physical activity of children and adolescents. Medcine & Science in Sports and Exercise. 2000;32:963 - 75.

34. Ferreira I, van der Horst K, Wendel-Vos W, Kremers S, van Lenthe FJ, Brug J. Environmental correlates of physical activity in youth - a review and update. Obesity Reviews. 2006;8(2):129-54.

35. Crawford D, Cleland V, Timperio A, Salmon J, Andrianopoulos N, Roberts R et al. The longitudinal influence of home and neighbourhood environments on children's body mass index and physical activity over 5 years: the CLAN study. International Journal of Obesity 2010;34(7):1177-87. doi:10.1038/ijo.2010.57.

36. Hesketh K, Crawford D, Salmon J. Children's television viewing and objectively measured physical activity: associations with family circumstance. International Journal of Behavioral Nutrition and Physical Activity. 2006;3(1):36.

37. McMinn AM, Van Sluijs EMF, Wedderkopp N, Froberg K, Griffin SJ. Sociocultural Correlates of Physical Activity in Children and Adolescents: Findings From the Danish Arm of the European Youth Heart Study. Pediatric exercise science. 2008;20(3):319-32.

38. Riddoch C, Edwards D, Page A, Froberg K, Anderssen SA, Wedderkopp N et al. The European Youth Heart Study—Cardiovascular Disease Risk Factors in Children: Rationale, Aims, Study Design, and Validation of Methods. Journal of physical activity & health. 2005;2(1):115.
